# One Cycle of Concurrent Chemotherapy vs. Two Cycles of Concurrent Chemotherapy With Radiation Therapy in Patients With Limited-Stage Small Cell Lung Cancer

**DOI:** 10.3389/fonc.2021.785022

**Published:** 2022-01-24

**Authors:** Hao Yu, Jiaqi Zhang, Zhen Zhang, Youyou Wang, Guangying Xu, Liming Xu, Ningbo Liu, Lujun Zhao, Ping Wang

**Affiliations:** ^1^ Department of Radiation Oncology, Tianjin Medical University Cancer Institute and Hospital, National Clinical Research Center for Cancer, Key Laboratory of Cancer Prevention and Therapy, Tianjin’s Clinical Research Center for Cancer, Tianjin, China; ^2^ Department of Radiation Oncology (MAASTRO), GROW School for Oncology and Developmental Biology, Maastricht University Medical Centre, Maastricht, Netherlands; ^3^ Department of Radiation Oncology, Tianjin First Central Hospital, Tianjin, China

**Keywords:** LS-SCLC, prognosis, PCI, concurrent chemoradiotheraphy, propensity score (PS) matching (PSM)

## Abstract

**Background:**

The optimal number of concurrent chemotherapy cycles during thoracic radiotherapy (RT) in patients with limited stage-small cell lung cancer (LS-SCLC) is not well defined. The purpose of this study was to evaluate the impact of the number of concurrent chemotherapy cycles on prognosis of LS-SCLC.

**Material and Methods:**

Patients with LS-SCLC treated with concurrent chemo-radiotherapy from May 2008 to December 2020 in our hospital were retrospectively analyzed. The prescribed radiation dose was 60Gy administrated with conventional RT in 30 fractions within 6 weeks. The prognostic role of cycle number of chemotherapy administrated concurrently with RT were analyzed. All patients were followed up at one month after the treatment, then once every three months until two years after the treatment, and every six months thereafter. Propensity score matching (PSM) was performed to reduce confounding factors. The primary endpoint was overall survival (OS). Survival analysis was performed with Kaplan-Meier and multivariate analysis was performed with Cox regression model.

**Results:**

Among the 370 patients who received radical radiotherapy, 206 patients received concurrent chemo-radiotherapy and were included for the analysis. Multivariate analysis showed that stage and PCI were independent prognostic factors for OS. The median OS in patients who received one cycle and two cycles of chemotherapy concurrently with RT were 32.9 months and 31.6 months, respectively (P = 0.241). And the median PFS were 20.6 months and 18.4 months, respectively (P = 0.764). After PSM, no statistical differences in OS and PFS were observed between patients who received one cycle and those who received two cycles of concurrent chemotherapy.

**Conclusion:**

Two cycles of concurrent chemotherapy during RT were not necessarily superior compared to one cycle in LS-SCLC. The optimal cycle number of concurrent chemotherapy during RT needs to be further studied.

## Introduction

Combination of radiotherapy (RT) and chemotherapy are considered standard treatments for patients with limited-stage small cell lung cancer (LS-SCLC) who are in a good physical condition (1, 2). Patients can receive accelerated hyperfractionated radiotherapy (i.e., 30 times of total dose of 45Gy radiotherapy in 3 weeks) or conventional radiotherapy (In China, most of the LS-SCLC patients received 60Gy in 30 fractions of radiation in about 6 weeks) in combination with concurrent chemotherapy Patients who received accelerated hyperfractionated radiotherapy can only receive 1 cycle of concurrent chemotherapy within 3 weeks of radiotherapy; while patients who received conventional radiotherapy can receive 2 cycles of concurrent chemotherapy (3, 4). To the best of our knowledge, there is no research published so far on the optimal number of cycles of concurrent chemotherapy during radiotherapy. In order to explore the impact of the number of cycles of concurrent chemotherapy on the prognosis of LS-SCLC who received radical radiotherapy, we conducted this retrospective study.

## Materials and Methods

### Patient Selection

This is a single institutional retrospective study which was approved by our IRB and the informed consent was waived. Eligible criteria included: 1) patients diagnosed with histopathologically proved LS-SCLC (Limited stage: Stage I-III (T any, N any, M0) that can be safely treated with definitive radiation doses. Excludes T3-4 due to multiple lung nodules that are too extensive or have tumor/nodal volume that is too large to be encompassed in a tolerable radiation plan); 2) Patients who received concurrent chemo-radiotherapy for radical purpose from May 2008 to December 2020. The exclusion criteria included: 1) patients who received chemotherapy only, 2) patients who had received surgery, and 3) patients who received only palliative radiotherapy (total dose < 50Gy). All cases were re-staged according to the AJCC 7^th^ edition (2010) lung cancer TNM staging. Due to the small number of stage I and II cases, those patients were combined into stage I/II in the subsequent prognostic analysis.

### Treatment Strategy

All the patients included in the study received chemotherapy concurrently or sequentially with radiation therapy. The total number of chemotherapy cycles was 4 to 6 (median 5 cycles). The majority of patients received etoposide (100 mg/m^2^, from day 1 to day 3, or 100 mg, from day 1 to day 5) combined with cisplatin (60 mg/m^2^ to 75 mg/m^2^, d1, or 40 mg, d1-d3) or carboplatin (carboplatin: 300 mg/m2, d1). A few patients received paclitaxel combined with platinum chemotherapy or etoposide single-agent chemotherapy. Patients received each cycle of chemotherapy every 3 weeks.

All patients received conventional thoracic radiotherapy with a 6 MV linear accelerator, and 3D conformal radiotherapy (3D-CRT) or intensity-modulated radiation therapy (IMRT) planning were used. GTV (gross tumor volume) included imaging visible lesions including primary tumor and metastatic lymph nodes. The planning gross tumor volume (PTV_G_) was generated by uniformly expansion of the GTV by a 5 mm margin. Clinical target volume (CTV) was defined as the high-risk lymph nodal regions, including adjacent regions of involved lymph nodes and the ipsilateral hila and the GTV with a 5mm margin. Planning target volume (PTV) was generated with 5mm-10mm expansion to the CTV. The prescribed radiation dose was 60Gy to PTV_G_ with a fraction dose of 2.0Gy and 54Gy to the PTV with a fraction dose of 1.8Gy, 5 fractions per week. For patients whose PTV_G_ was not delineated, the prescribed radiation dose to PTV was 60Gy. Normal organ constraints included: maximum dose to spinal cord < 45 Gy; lung V20 ((i.e., the percentage of total lung volume receiving ≧ 20Gy) < 28%, Mean lung dose < 17 Gy; esophagus V50 < 50%, Dmax < 60Gy; heart V30 < 40% (5).

The patients were divided into one cycle concurrent chemotherapy (1 cycle group) or two cycles concurrent chemotherapy (2 cycles group) groups based on the number of concurrent chemotherapy cycles during the course of thoracic RT. And they were further divided into early RT or late RT groups based on the time thoracic RT was initiated. Early RT included patients who received RT before the beginning of the 4^th^ cycle of chemotherapy and late RT included patients who received RT after the beginning of the 4^th^ cycle of chemotherapy (6, 7).

### Follow-Up and Statistical Analysis

All patients were followed up at one month after the treatment, then once every three months until two years after the treatment, and every six months thereafter. The follow-up examination included evaluating symptoms and signs, chest CT, brain MRI, and bone scan or PET/CT examination if necessary. The primary endpoint was overall survival (OS), which was calculated from the start of treatment to the time of death due to any cause or the time of the last follow-up. The second endpoint was progression-free survival (PFS), which was defined as the time from the beginning of treatment to the time of disease recurrence or metastasis or the date of the last follow-up. Treatment-related toxicity was evaluated according to CTCAE (Common Terminology Criteria for Adverse Events) 4.0 standards.

Propensity score matching (PSM) was performed between 1 cycle group and 2 cycles group to reduce potential bias in this study. The clinically significant variables were chosen as matching factors in PSM. The match tolerance (caliper) was set at 0.05, and 1:1 nearest neighbor matching was performed.

SPSS 23.0 software was used for statistical analysis and PSM. Categorical data comparison between groups was performed by chi-square test. Kaplan-Meier method was used to calculate OS and PFS. Log-Rank method was used to compare survival differences between groups, and multivariate survival analysis was performed by Cox regression model.

## Results

### Patient Characteristics

A total of 370 patients received radical radiotherapy in our hospital from May 2008 to December 2020. Among them, 206 patients received concurrent chemo-radiotherapy and were included in this analysis. All the patients received induction chemotherapy, with a median of 3 cycles (range:1-7 cycles), and 152 patients received consolidation chemotherapy with a median of 2 cycles (range: 2-6 cycles). Ninety-eight patients received one cycle of concurrent chemotherapy and 108 patients received two cycles of concurrent chemotherapy.

The concurrent chemotherapy regimens were all EP or EC regimens. Patients who received only one cycle of concurrent chemotherapy were tend to be older and with poorer performance status compared with those who received two cycles of chemotherapy. The characteristics of the patients in the two groups are listed in **Table 1**.

**Table 1:** Clinical characteristics of patients in 1 cycle and 2 cycles groups before and after propensity score matching.

Characteristic		Pre-propensity score matching (n/%)			Post propensity score matching (n/%)		
		1 Cycle (n = 98)	2 cycles (n = 108)	P value	1 Cycle (n = 76)	2 cycles (n = 76)	P value
Gender	Male	73	82	0.872	55	57	0.854
	Female	25	26		21	19	
Age	<65	67	87	0.054	54	57	0.715
	≥65	31	21		22	19	
Weight Loss	Yes	72	78	0.876	20	20	1.000
	No	26	30		56	56	
Smoking Status	Yes	73	74	0.359	56	55	1.000
	No	25	34		20	21	
KPS	≥80	73	93	0.052	59	61	0.843
	<80	25	15		17	15	
Clinical Stage	I+II	19	20	0.874	16	15	1.000
	III	79	88		60	61	
Timing of RT	Early	85	102	0.089	69	70	1.000
	Late	13	6		7	6	
PCI	No	37	61	0.008	42	39	0.854
	Yes	61	47		34	37	

After chemo-radiotherapy, CR or PR was observed in 157 patients (76.2%) and SD or PD was observed in 49 patients (23.8%). PCI was administrated in 108 patients (89 in CR or PR response group and 19 in SD or PD response group) based on physician’s recommendation and patients’ acceptance. Information about second line treatment were observed in thirty-six patients (28 in 1 cycle group and 8 in 2 cycle group) after disease progress, with chemotherapy of irinotecan or docetaxel regimens.

### Prognostic Factors for OS

The median follow-up of survival patients was 22.4 months by the last follow-up on Aug. 25^th^ 2021. The median OS, 1-year and 3-year OS were 32.9 months, 83.0% and 20.9% respectively. The median PFS, 1-year and 3-year PFS were 18.6 months, 56.3%, and 15.1% respectively. Univariate analysis showed that stage, age and PCI were all prognostic indicators for OS. Multivariate regression analysis showed that only clinical stage and PCI are independent prognostic factors affecting OS. Early stage, administration of PCI are significantly correlated with prolonged OS (**Table 2**).

**Table 2:** Result of univariate analysis and multivariable analysis.

	Univariate analysis			Multivariable analysis		
	HR	95% CI	P value	HR	95% CI	P value
Gender	0.807	0.506 - 1.288	0.369	0.873	0.543-1.402	0.573
(Male vs. Female)						
Age	1.503	0.989-2.283	0.056	1.26	0.811-1.957	0.303
(< 65 vs. ≥ 65)						
KPS	1	0.622-1.608	0.999	1.706	0.666-1.736	0.766
(≥ 80 vs. < 80)						
Clinical stage	2.96	1.49-5.88	0.002	2.889	1.452-5.749	0.003
(I-II vs. III)						
Timing of RT	1.381	0.770-2.478	0.278	1.315	0.722-2.396	0.37
(Early vs. Late)						
PCI	0.611	0.408-0.915	0.017	0.684	0.445-1.049	0.082
(No vs. Yes)						

HR, hazard ratio; CI, confidence interval; RT, radiotherapy; CRT, chemoradiotherapy; KPS, Karnofsky Performance Score.

### Impact of the Number of Cycles of Concurrent Chemotherapy on Prognosis

**Figure 1:**
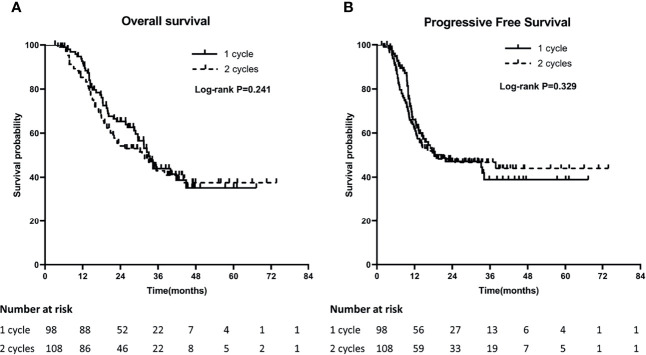
Comparison of Overall survival (OS) **(A)** and Progression-Free Survival (PFS) **(B)** between 1 cycle and 2 cycle groups.

The median OS of patients with 1 cycle and 2 cycles of concurrent chemotherapy were 32.9 months and 31.6 months, and the 1-year and 3-year OS were 89.8% and 22.4% versus 79.6% and 20.4%, respectively (P = 0.241). The median PFS was 20.6 months and 18.4 months, and the 1-year and 3-year PFS were 57.1% and 13.3% versus 56.5% and 17.6%, respectively (P = 0.764). (**Figures 1A, B** **)**.

**Figure 2:**
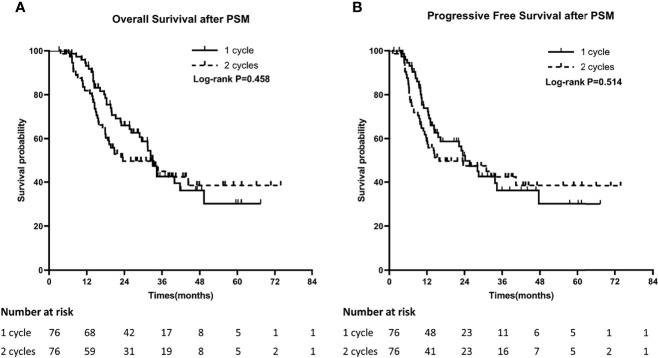
Comparison of Overall survival (OS) **(A)** and Progression-Free Survival (PFS) **(B)** between 1 cycle and 2 cycle groups after PSM.

In order to minimize the bias of the retrospective study, we performed propensity score matching with age, gender, KPS, smoking status, weight loss, disease stage, timing of RT and PCI as the matching factors (**Table 1**). After PSM, 76 patients were included in each of the groups. The median OS in one cycle and two cycles groups were 33.0 months and 23.5 months, and the 1-year and 3-year OS were 89.5% and 22.4% versus 77.6% and 25.0%, respectively (P=0.458). The median PFS in the two groups were 24.1 months and 16.1 months, and the 1-year and 3-year PFS were 63.2% and 14.5% versus 53.9% and 21.0%, respectively (P=0.514) (**Figures 2A, B** **)**.

### Adverse Events in Different Groups

The incidence of treatment related adverse events were similar in the two groups. In patients who received one cycle of concurrent chemotherapy, 19 patients (19.4%) developed ≧ grade 3 adverse events, and 15 patients (14.7%) in 2 cycles of concurrent chemotherapy group (P=0.988) (**Table 3**).

**Table 3:** Comparison of adverse events in patients with different concurrent chemotherapy cycles.

Adverse Events	1 cycle concurrent CRT (98 cases)				2 cycles concurrent CRT (108 cases)			
	Grade 1	Grade 2	Grade 3	Grade 4	Grade 1	Grade 2	Grade 3	Grade 4
Radiation pnemonia	5 (5)	6 (6)	2 (2)	–	15 (15)	6 (6)	2 (2)	–
Radiation	2 (2)	15 (15)	–	–	7 (7)	16 (16)	3 (2)	–
Esophagtis								
Leukopenia	5 (5)	23 (23)	6 (6)	4 (4)	18 (17)	24 (23)	8 (8)	–
Thrombocytopenia	3 (3)	5 (2)	3 (3)	2 (2)	8 (7)	1 (1)	1 (1)	–
Gastrointestinal adverse Events	7 (7)	6 (6)	2 (2)	–	11 (11)	5 (5)	1 (1)	–

## Discussion

Our single-center retrospective study showed that disease stage and PCI are independent factors for OS and PFS in LS-SCLC, and the number of concurrent chemotherapy cycles has no significant impact on the prognosis of LS-SCLC. After PSM, Survival analysis still showed that patients who received one cycle of concurrent chemotherapy had similar OS and PFS outcomes compared with two cycle groups.

Generally, patients with SCLC were classified into limited stage and extensive stage based on the probability of being treated with definitive radiation doses safely. However, LS-SCLC can be further classified into stage I, stage II, and stage III based on the TNM staging system. And it was reported that TNM staging predicts treatment outcomes more accurately (8, 9). Our results emphasized the importance of using TNM staging system in SCLC again.

Though with some controversy after the publication of Takahashi’s study (10–15), PCI is still recommended in many guidelines (16–18). However, the application of PCI is not consistently accepted by doctors or patients. Only about 70% of patients with LS-SCLC received PCI in M. D. Cancer center (13) and in the Princess Margaret Cancer Centre (10), and 57% of the patients with CR or PR response after chemoradiotherapy received PCI in Nakamura’s report form Japan (14). In the present study, only 52.4% of the patients received PCI. Patients who received PCI had better response to chemo-radiotherapy, and hence with better OS. The administration of PCI in patients with LS-SCLC should be used after fully communication with every individual patient.

Concurrent chemo-radiotherapy is recommended as the standard treatment for LS-SCLC (19). For patients who received accelerated hyperfractionated radiotherapy, only one concurrent chemotherapy cycle can be used during the 3 weeks period of radiotherapy. However, for patients who received conventional fractionated radiation, 2 cycles of concurrent chemotherapy can be given in the 6-7 weeks period of RT. But some patients cannot tolerate two cycles of concurrent chemotherapy during the course of RT. Whether it is necessary to receive two cycles of chemotherapy in patients who received conventional fractionated RT is unclear. Our retrospective study found that the number of cycles has no significant impact on the prognosis. Our study suggested that in patients who cannot tolerate two cycles of chemotherapy during the course of RT don’t have to receive two cycles of chemotherapy. One cycle of chemotherapy can obtain similar OS and PFS.

There are many factors affecting the choice of one cycle or two cycles of concurrent chemotherapy. Patients with older age and poor performance most likely cannot tolerate concurrent chemotherapy well. In our study, it was found that the patients who received just one cycle instead of two cycles of concurrent chemotherapy were older, had a worse PS, received more induction chemotherapy before RT, and had worse tolerance to the treatment. In addition, less patients in this group received PCI after chemoradiotherapy. Generally, older age, poor PS, late RT, no PCI are all negatively correlated with OS (20–23), and reduction of treatment intensity also have a negative impact on survival. Thus those who received only one cycle of concurrent chemotherapy should had a worse OS. However, in our study, though with more unfavorable factors, they had a similar survival outcome with those who received two cycles of concurrent chemotherapy.

The present study has some limitations due to the nature of retrospective analysis. First, the distribution of clinical characteristics were not balanced very well between the two groups. And the treatment strategy including induction chemotherapy, timing of RT, consolidation chemotherapy, PCI for each patient were not consistent in each group, and might affect treatment outcomes. Though we used PSM to reduce the impact of the confounding factors, the bias cannot be completely eliminated.

## Conclusion

To the best of our knowledge, this is the first report about the impact of concurrent chemotherapy cycle number on prognosis in patients with LS-SCLC. Based on our single center retrospective analysis, it was found that there are no significant differences in OS and PFS between patients who received one cycle and those who received two cycles of concurrent chemotherapy. Patients with LS-SCLC don’t have to receive two cycles concurrent chemotherapy during the course of RT if they cannot tolerate it very well due to older age or poor performance. Large prospective randomized controlled study is warranted to further verify our findings.

## Data Availability Statement

The raw data supporting the conclusions of this article will be made available by the authors, without undue reservation.

## Ethics Statement

The studies involving human participants were reviewed and approved by Medical Ethics Committee of Tianjin Cancer Hospital. Written informed consent for participation was not required for this study in accordance with the national legislation and the institutional requirements.

## Author Contributions

HY, PW, and LZ contributed to conception and design of the study. HY, JZ, and ZZ organized the database. YW performed the statistical analysis. HY and GX wrote the first draft of the manuscript. HY, NL, and LX wrote sections of the manuscript. All authors contributed to manuscript revision, read, and approved the submitted version.

## Conflict of Interest

The authors declare that the research was conducted in the absence of any commercial or financial relationships that could be construed as a potential conflict of interest.

## Publisher’s Note

All claims expressed in this article are solely those of the authors and do not necessarily represent those of their affiliated organizations, or those of the publisher, the editors and the reviewers. Any product that may be evaluated in this article, or claim that may be made by its manufacturer, is not guaranteed or endorsed by the publisher.

## Abbreviations

LS-SCLC, limited-stage small cell lung cancer; OS, Overall survival; PFS, Progression-free survival; PSM, Propensity score matching; PCI, Prophylactic Cranial Irradiation; RT, Radiotherapy.
